# Alloying design of biodegradable zinc as promising bone implants for load-bearing applications

**DOI:** 10.1038/s41467-019-14153-7

**Published:** 2020-01-21

**Authors:** Hongtao Yang, Bo Jia, Zechuan Zhang, Xinhua Qu, Guannan Li, Wenjiao Lin, Donghui Zhu, Kerong Dai, Yufeng Zheng

**Affiliations:** 1 Beijing Advanced Innovation Center for Materials Genome Engineering & Department of Materials Science and Engineering, College of Engineering, Peking University, Beijing, 100871 China; 2 Department of orthopaedic surgery, Shanghai Key Laboratory of Orthopedic Implants, Shanghai Ninth People’s Hospital, Shanghai Jiaotong University School of Medicine, Shanghai, 200011 China; 3 R&D Center, Lifetech Scientific (Shenzhen) Co Ltd, Shenzhen, 518057 China; 4 Department of Biomedical Engineering, College of Engineering and Applied Sciences, Stony Brook University, Stony Brook, NY 11794-5281 USA; 5 International Research Organization for Advanced Science and Technology, Kumamoto University, 2-39-1 Kurokami, Chuo-Ku, Kumamoto, 860-8555 Japan

**Keywords:** Fracture repair, Biomaterials, Metals and alloys

## Abstract

Magnesium-based biodegradable metals (BMs) as bone implants have better mechanical properties than biodegradable polymers, yet their strength is roughly less than 350 MPa. In this work, binary Zn alloys with alloying elements Mg, Ca, Sr, Li, Mn, Fe, Cu, and Ag respectively, are screened systemically by in vitro and in vivo studies. Li exhibits the most effective strengthening role in Zn, followed by Mg. Alloying leads to accelerated degradation, but adequate mechanical integrity can be expected for Zn alloys when considering bone fracture healing. Adding elements Mg, Ca, Sr and Li into Zn can improve the cytocompatibility, osteogenesis, and osseointegration. Further optimization of the ternary Zn-Li alloy system results in Zn-0.8Li-0.4Mg alloy with the ultimate tensile strength 646.69 ± 12.79 MPa and Zn-0.8Li-0.8Mn alloy with elongation 103.27 ± 20%. In summary, biocompatible Zn-based BMs with strength close to pure Ti are promising candidates in orthopedics for load-bearing applications.

## Abstract (web summary)

Biodegradable implants are of great interest in orthopaedic applications but have been limited by low mechanical strength. Here, the authors examine systematically in detail the strengthening of biodegradable zinc by alloying with beneficial elements using mechanical, biodegradability and biocompatibility testing.

## Introduction

To address the clinical problems associated with stress shielding and secondary surgery, biodegradable materials provide an alternative option to permanent materials in orthopedics. Biodegradable polymers like poly(glycolic acid) (PGA), poly(l-lactic acid) (PLLA) and poly(lactic acid-co-glycolic acid) (PLGA) have been approved by Food and Drug Administration (FDA) for clinical use as bone screws, nails and pins, suture anchors and meniscal darts, etc^1,2^. Meanwhile, bone screws made of Mg-Y-RE-Zr^3^ and Mg-Ca-Zn^4^ alloys have been approved by Conformité Européene (CE) and the Korea Food and Drug Administration (KFDA) in 2013 and 2015, respectively. Additionally, high purity Mg screws used for fixation of autologous bone grafts or bone fractures are undergoing clinical trials in China^5^. However, there is still a great gap between the mechanical strength of biodegradable materials like polymers^1,2^ and Mg alloys^6^ (UTS < 350 MPa) and traditional metallic materials like cobalt chromium alloys^7^, 316 L stainless steel and titanium-based alloys^8^ (UTS > 500 MPa). Therefore, the clinical use of biodegradable implants has been limited to non or low load-bearing applications such as fixation of small bone and cancellous fragments, meniscus repair and soft tissue fixation^2–4^.

Recent advances in biodegradable Zn alloys have developed novel alloy systems such as Zn–Mg^9,10^ (UTS 155–455 MPa) and Zn–Li^11^ (UTS 360–560 MPa) alloys with outstanding mechanical strength. Zinc plays an essential role in bone metabolism. Zinc supplementation stimulates osteoblast bone formation, meanwhile, inhibiting osteoclast differentiation and results in increased bone strength^12,13^. Therefore, biodegradable Zn alloys appear to exhibit distinct advantages over biodegradable polymers and Mg alloys in orthopedic applications. However, current research of biodegradable Zn alloys has focused on material aspects. A wide range of composition has been explored without a clear design principle^14,15^. For bone implants, limited publications have focused on only three alloy systems including Zn–Mg, Zn–Ca, and Zn–Sr alloys^9,16–19^. Moreover, in vitro methodology has been performed predominantly in these studies. According to the research experience from biodegradable Mg alloys^20^, the existence of a great discrepancy between in vitro and in vivo data makes it hard to predict the real performance of implants under physiological conditions. Therefore, it is necessary to combine the findings from both in vitro and in vivo to instruct the alloying design for Zn alloys as bone implants. To establish binary Zn alloy systems, eight beneficial elements for bone health including Mg^21^, Ca^22^, Sr^23,24^, Li^25,26^, Mn^27^, Fe^28,29^, Cu^30,31^, and Ag^32^ were selected as alloying elements adding into zinc. For elements with low or no solubility in zinc (Mg, Ca, Sr, Li, Mn, and Fe), alloy contents were set at 0.1, 0.4, and 0.8 wt%. Cu and Ag were added at 0.4, 0.8, and 2.0 wt% due to their relatively high solubility in zinc.

In this study, binary Zn alloys were screened by in vitro tests in the context of mechanical property, corrosion behavior, and cellular response. Alloy composition with superior performance in each alloy system was further implanted into rat femur for in vivo evaluation. Ternary Zn–Li–X (X = Mg or Mn) alloys were designed as an optimization based on the Zn–Li alloy system. As a result, the alloying design strategy for Zn alloys as bone implants is proposed regarding the mechanical property, biodegradation, and biocompatibility. The present study may provide guidance on the future clinical prospects of Zn-based materials in orthopedic applications.

## Results

### Microstructure analysis

**Fig. 1:**
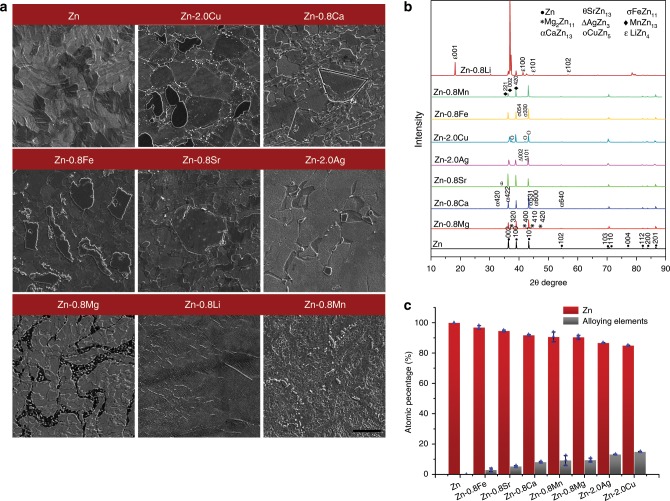
Microstructure analysis of pure Zn and binary Zn alloys at the highest alloy contents. **a** SEM images. Scale bar, 10 μm. **b** X-ray diffraction (XRD). **c** Chemical composition analysis of intermetallic phases in binary Zn alloys. Error bars indicate mean ± standard deviation (*n* = 3). Source data are provided as a Source Data file.

Supplementary Fig. 1 and Fig. 1a presents SEM micrographs showing the microstructure of as-extruded pure Zn and binary Zn alloys at different alloy contents. The microstructure of pure Zn was refined after extrusion with a grain size of less than 10 μm. Distinct features were observed in different alloy systems compared with pure Zn. At low alloy contents (Supplementary Fig. 1), although X-ray diffraction only identified the intermetallic phases in Zn-0.1Li and Zn-0.1Ca alloys, intermetallic phases could be observed in Zn-0.1Mg, Zn-0.1Sr, and Zn-0.1Fe alloys as well due to their limited or no solubility in Zn (Mg: 0.1 wt% at 364 °C, Sr and Fe: no solubility in Zn). In contrast, Zn-0.1Mn, Zn-0.4Cu, and Zn-0.4Ag alloys consisted of single-phase solid solutions as a result of their relatively high solubility in Zn (Mg: ~0.8 wt% at 405 °C, Cu: 2.75 wt% at 425 °C, Ag: 8 wt% at 431 °C). At high alloy contents (Fig. 1), the volume fraction of intermetallic phases increased in all alloy systems. The feature of intermetallic phases in different alloy systems could be roughly divided into two categories. The first category is the case where the size of the second phase was larger than the grain size of surrounding Zn-rich matrix, such as in Zn-2.0Cu, Zn-0.8Ca, Zn-0.8Fe, and Zn-0.8Sr. The second is the opposite case where the size of the second phase was finer than the Zn grain, such as in Zn-2.0Ag, Zn-0.8Mg, Zn-0.8Li, and Zn-0.8Mn. X-ray diffraction identified the characteristic peaks of intermetallic phases formed between Zn and alloying elements (Fig. 1b). Generally, biphasic microstructures were typical in binary Zn alloy systems with relatively high alloy contents. Quantitively analysis illustrated that the intermetallic phases in Zn-2.0Ag (AgZn_3_), Zn-2.0Cu (CuZn_5_), and Zn-0.8Mg (Mg_2_Zn_11_) contained relatively higher contents of alloying elements than other groups (Fig. 1c).

### Mechanical performance

**Fig. 2:**
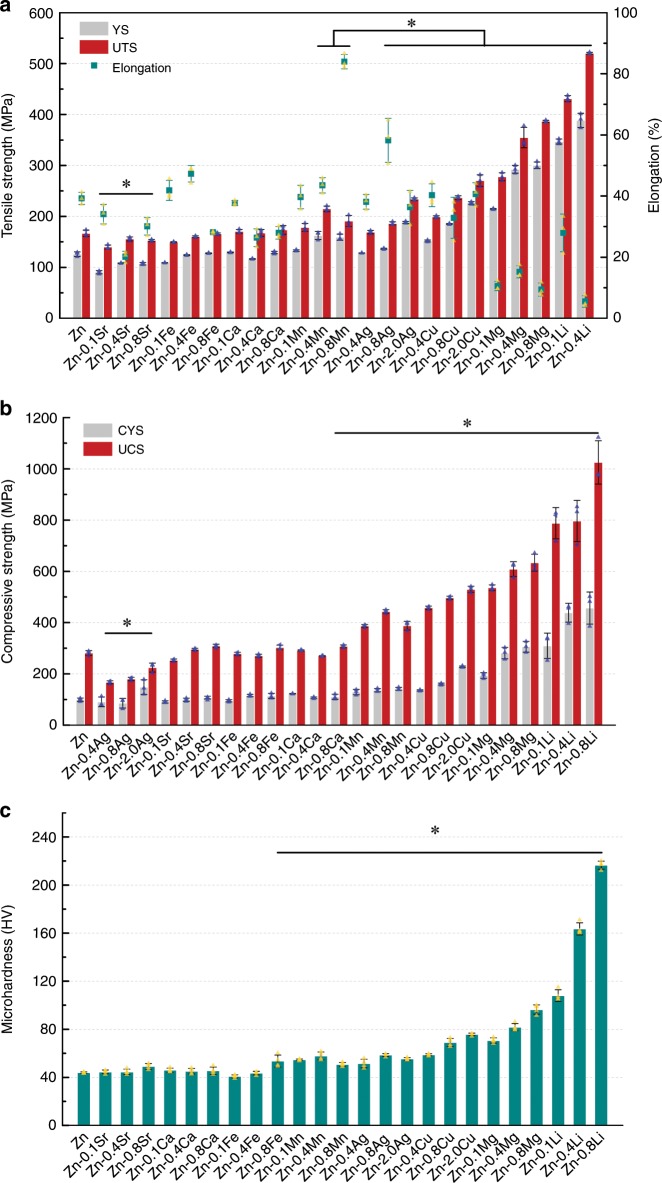
Mechanical performance of pure Zn and binary Zn alloys. **a** Tensile test. **b** Compression test. **c** Microhardness. Error bars indicate mean ± standard deviation (tensile and compressive tests, *n* = 3; Microhardness, *n* = 4, independent samples). *P < 0.05 by one-way ANOVA with Tukey’s post hoc test, compared with pure Zn. Source data are provided as a Source Data file.

The mechanical properties were evaluated by tensile test, compression test, and microhardness. As shown in Fig. 2a, dramatic differences were found in tensile strength and elongation when adding different elements even at minor additions. Among them, Li, Mg, Cu, Ag, and Mn demonstrated strengthening effects on pure Zn. Moreover, the strength is further improved with element contents. Li played the most significant role in strengthening the Zn matrix. Addition of 0.1 wt% Li increased the ultimate tensile strength (UTS) of pure Zn from 166.79 ± 6.36 MPa to 431.27 ± 5.89 MPa (*n* = 3). Zn-0.4Li alloy reached the maximum UTS of 520.36 ± 1.83 MPa *(n* = 3). We failed to record the strength of the Zn-0.8Li alloy because it fractured before yielding. However, the ductility declined significantly with Li and Mg additions. Zn–Cu, Zn–Ag, and Zn-Mn alloys maintained superior ductility compared with pure Zn. What stands out is that the addition of 0.8 wt% Ag and Mn even improved the elongation to failure of pure Zn from 39.22 ± 2% to 58.22 ± 7.18% and 83.96 ± 2.36% (*n* = 3), respectively. In contrast, alloying with Ca, Fe, and Sr up to 0.8 wt% appeared to have little influence on the strength of pure Zn. Additionally, their ductility dropped with contents. The influence of alloying elements on compressive strength (Fig. 2b) and microhardness (Fig. 2c) was similar to that on tensile strength. Surprisingly, adding Ag caused a decrease in compressive strength indicating an opposite role of Ag in tensile and compressive behavior of Zn.

### Corrosion behavior

**Fig. 3:**
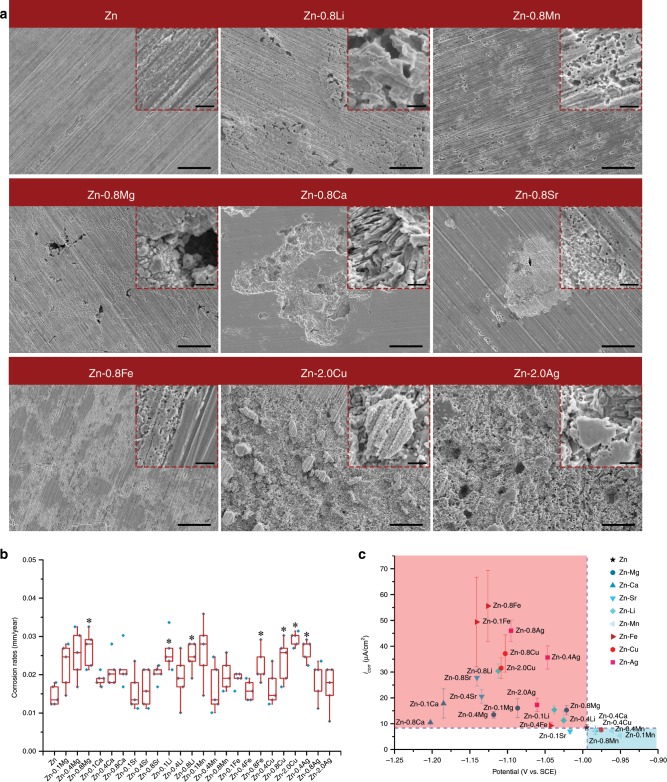
In vitro corrosion behavior of pure Zn and binary Zn alloys. **a** Corrosion morphology of selected materials after removal of corrosion products, intermetallic phases are marked by white arrows. Scale bar, 20 μm in low magnification, 2 μm in the inserts. **b** Corrosion rates calculated on weight loss (*n* = 5, independent samples). *P < 0.05 by one-way ANOVA with Tukey’s post hoc test, compared with pure Zn. For box-whisker plots, box edges correspond to 25th and 75th percentiles, lines inside the box correspond to 50th percentiles, and whiskers include minimum and maximum of all data points. **c** Corrosion current density verses corrosion potential based on results of electrochemical test. Error bars indicate mean ± standard deviation (*n* = 3, independent samples). Source data are provided as a Source Data file.

Immersion test and electrochemical test were utilized to assess the corrosion behavior of binary Zn alloys with pure Zn as control. Supplementary Fig. 2 provides the representative corrosion morphologies of samples after immersion in SBF for 30 days. Pure Zn was covered by an intact corrosion layer with tiny precipitates on the surface. This layer was relatively thin with the scratches left by grinding could be clearly seen. In contrast, the corrosion morphologies of binary Zn alloys were similar to that of pure Zn except Zn-0.8Ca alloy. A thicker corrosion layer formed on Zn-0.8Ca alloy. Precipitates in a much larger size were observed on the surface of the corrosion layer. After the removal of corrosion products, distinct features were observed in different alloy systems (Fig. 3a). The morphology of pure Zn was complete. In contrast, galvanic corrosion was clearly seen in binary Zn alloys on a micro scale. Corrosion took place preferentially in the intermetallic phases in Zn-0.8Li, Zn-0.8Mn, Zn-0.8Mg, Zn-0.8Ca, and Zn-0.8Sr alloys. Among them, corrosion pits were several microns in size and distributed more uniformly in Zn-0.8Li and Zn-0.8Mn alloys, whereas the pits were much larger in Zn-0.8Ca and Zn-0.8Sr alloys. As for Zn-0.8Fe, Zn-2.0Cu, and Zn-0.8Ag alloys, their intermetallic phases were almost intact with surrounding Zn matrix being severe corroded. The second phases protruded apparently in the corroded area in Zn-2.0Cu and Zn-0.8Ag alloys while less difference was seen in Zn–Fe alloy. The corrosion rates calculated on weight loss are presented in Fig. 3b. Generally, alloying enhanced the corrosion rate of pure Zn to varying degrees. Corrosion rates of samples ranged from 0.014 ± 0.003 to 0.030 ± 0.001 mm year^−1^ (*n* = 5). In addition, there was an increasing trend in corrosion rates with alloy contents except for Zn–Mn and Zn–Ag systems. To further depict the influence of alloy elements on corrosion behavior, the corrosion current density versus corrosion potential is shown in Fig. 3c and Supplementary Table 3. In general, there was a negative shift in corrosion potential and an increase in corrosion current density in binary Zn alloys except for the Zn–Mn alloy system compared with pure Zn. Among them, Fe, Cu, and Ag played a more significant role in accelerating corrosion than Mg, Ca, Sr, and Li. Mn showed little influence on the corrosion behavior of pure Zn.

### Cytocompatibility with MC3T3-E1 cells and HUVEC cells

**Fig. 4:**
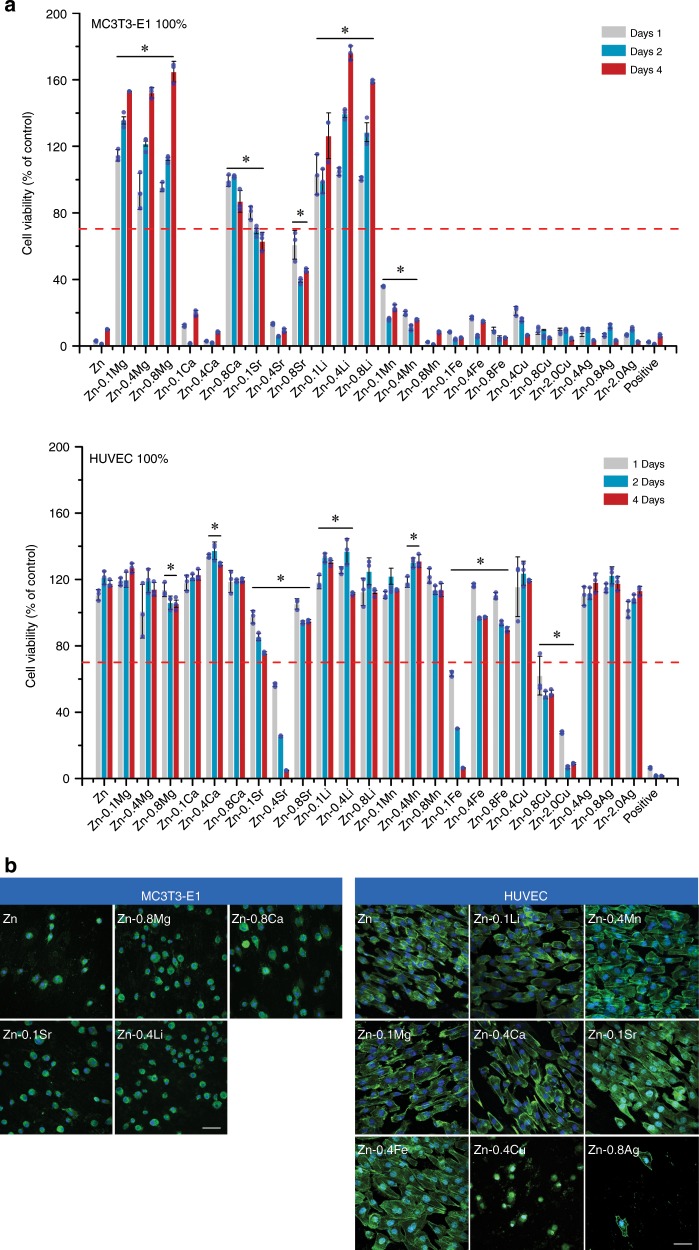
Cytocompatibility of pure Zn and binary Zn alloys. **a** Cell viability of MC3T3-E1 cells and HUVEC cells cultured with 100% extracts. Error bars indicate mean ± standard deviation (*n* = 3, independent samples). *P < 0.05 by one-way ANOVA with Tukey’s post hoc test, compared with pure Zn. The red dashed line indicates the cut-off between non-toxic and toxic responses according to ISO 10993-5. **b** Fluorescent images of attachment of MC3T3-E1 cells and HUVEC cells on selected sample surfaces at 12 h. Cell nucleus: DAPI, F-actin: FITC. Scale bar, 50 μm. Source data are provided as a Source Data file.

Osteogenesis and angiogenesis play pivotal roles in skeletal development and bone fracture healing. Therefore, Mouse Osteoblastic Cells (MC3T3-E1) and Human Umbilical Vein Endothelial Cells (HUVECs) were used to evaluate the cytocompatibility of binary Zn alloys with pure Zn as control. Figure 4a presents the cell viability of MC3T3-E1 and HUVEC cells cultured in 100% material extracts. What is striking about the data is that only Zn–Mg alloy and Zn–Li alloy extracts promoted the proliferation of MC3T3-E1 cells significantly. Whereas pure Zn and other binary Zn alloys exhibited severe cytotoxicity except for Zn-0.8Ca and Zn-0.1Sr alloys. After one-fold dilution, no toxicity was found in all groups (Supplementary Fig. 3a). In contrast, HUVEC cells showed better performance on 100% material extracts (Fig. 4b). Promotion in proliferation was found in pure Zn, Zn–Mg, Zn–Ca, Zn–Li, Zn–Mn, and Zn–Ag alloy groups while only Zn-0.4Sr, Zn-0.1Fe, Zn-0.8Cu, and Zn-2.0Cu showed cytotoxicity on HUVEC cells over time. In order to examine the cell morphology, materials with good cytocompatibility were selected for cell direct contact (Fig. 4b). After 12 h of culture on the sample surface, MC3T3-E1 cell displayed a round and unhealthy shape. The F-actin expression was limited and barely distinct from the DAPI signature. In contrast, HUVEC cells exhibited an elongated shape with F-actin filaments extending to the sample surface in all groups except for Zn-0.4Cu and Zn-0.8Ag alloys. Additionally, more F-actin was distributed at the cell edge. Ion concentrations of released Zn and alloy elements in the culture medium were detected and shown in Supplementary Fig. 3b. There was a decline of Zn ion concentrations in Zn–Ca and Zn–Li alloys compared with pure Zn. In contrast, an apparent increase in Zn ion concentration was seen in Zn–Fe, Zn–Cu, and Zn–Ag alloys. As for alloy elements, there was a rise in their ion concentrations compared with control. Among them, Ca ion increased about ~10 μg ml^−1^, followed by Li ion and Mg ion. The increase in ion concentration was less than 1 μg ml^−1^ in other alloy elements.

### In vivo degradation behavior

**Fig. 5:**
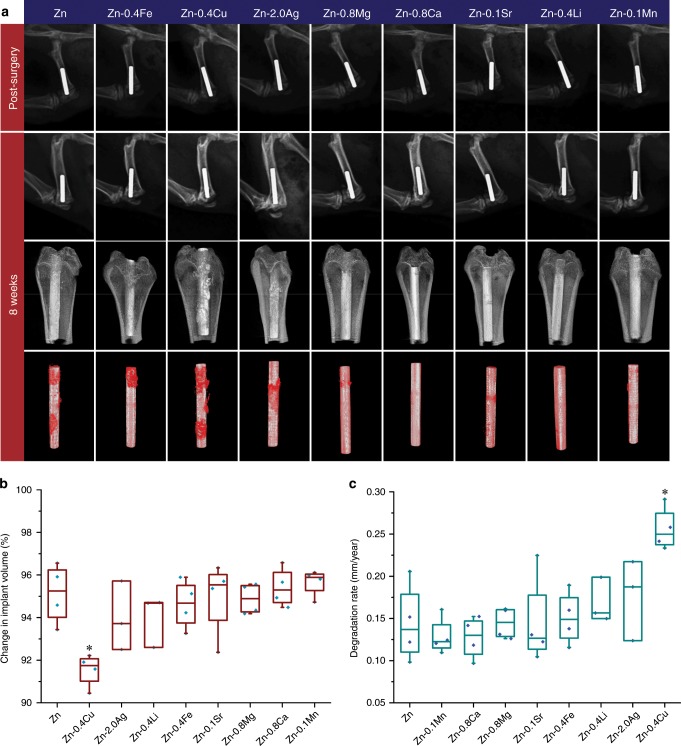
Micro-CT analysis on degradation behavior of implants. **a** Radiographs of implants in rat femurs. Post-surgery (first row), 8 weeks after implantation (second row), 8 weeks 3D reconstructions with bone tissue (third row) and 8 weeks 3D reconstructions without bone tissue (fourth row). Degradation products are marked in red. **b** Change in implant volume (*n* = 4 independent samples). **c** In vivo degradation rates (*n* = 4, independent samples). For box-whisker plots, box edges correspond to 25th and 75th percentiles, lines inside the box correspond to 50th percentiles, and whiskers include minimum and maximum of all data points. *P < 0.05 by one-way ANOVA with Tukey’s post hoc test, compared with Zn. Source data are provided as a Source Data file.

Binary Zn alloys with the best cytocompatibility in each alloy system were chosen for the animal test with pure Zn as control. A rat femur model was utilized to assess the in vivo performance of binary Zn alloys. Figure 5a shows the radiographs and reconstructed Micro-CT 3D image of implants post-surgery and at 8 weeks after implantation. All implants displayed distinct X-ray profiles, indicating their excellent radiopacity. Radiographs found no gas shadow in the femoral condyle and bone marrow cavity adjacent to the implants at the selected time points. No obvious degradation was detected for all implants at 8 weeks. Interestingly, the cortical bone around the implants became thicker with higher radiographic density over time, indicating the circumferential osteogenesis. All the implants demonstrated good biocompatibility with no signs of osteolysis, deformity or dislocation. Three-dimensional images showed new bone formation and direct contact between new bone and implants at 8 weeks. To evaluate the in vivo degradation behavior of implants, their metallic parts with degradation products were reconstructed. Generally, all the implants maintained their integrity at 8 weeks. Among them, pure Zn, Zn-0.4Fe, Zn-0.4Cu, and Zn-2.0Ag alloy implants displayed a localized degradation mode with local accumulation of products. In contrast, degradation of Zn-0.8Mg, Zn-0.8Ca, Zn-0.1Sr, Zn-0.4Li, and Zn-0.1Mn was more uniform on a macro scale. Volume change and degradation rate of implants were quantitatively measured and shown in Fig. 5b, c. At 8 weeks, the volume of pure Zn implant dropped to 95.12 ± 1.39% (*n* = 4) and its degradation rate was 0.14 ± 0.05 mm year^−1^ (*n* = 4). By contrast, Zn-0.4Cu alloys displayed a significant higher degradation rate of 0.26 ± 0.03 mm year^−1^ (*n* = 4). Moreover, accelerated degradation was observed in Zn-2.0Ag, Zn-0.4Li, Zn-0.4Fe, and Zn-0.8Mg alloys while Zn-0.1Mn, Zn-0.8Ca, and Zn-0.8Sr alloys exhibited similar degradation rates to that of pure Zn.

**Fig. 6:**
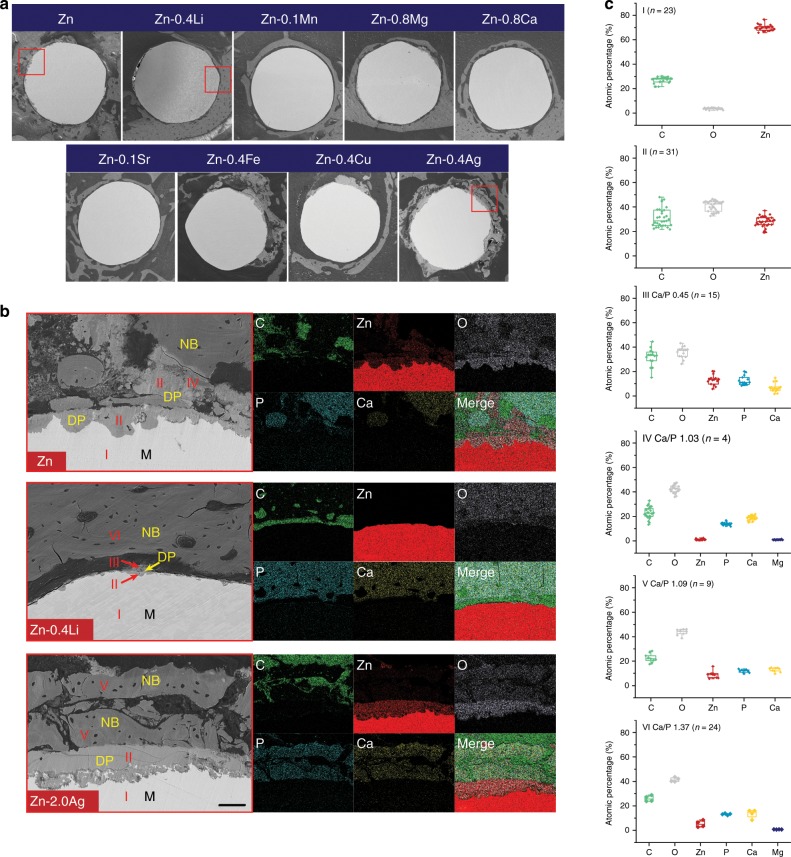
Cross sectional analysis on degradation behavior of implants. **a** Cross sectional images of implants. Scale bar, 0.5 mm. **b** Magnification of red rectangles in a with representative bone-implant interface and corresponding elemental mappings. NB: new bone; DP: degradation products; M: matrix. Scale bar, 50 μm. **c** Chemical compositions of marked regions in **b** (*n* values are shown in the figures, independent samples). For box-whisker plots, box edges correspond to 25th and 75th percentiles, lines inside the box correspond to 50th percentiles, and whiskers include minimum and maximum of all data points. Source data are provided as a Source Data file.

To further understand the degradation of Zn implants, the bone–implant interface was examined based on SEM and EDS on a micro level. Figure 6a presents the representative cross-sections containing both implants and surrounding tissue. The remaining metallic implants were visible as spherical features surrounding by degradation products and tissue. By matching the Micro-CT results, implants could be divided into two categories based on their degradation morphology. The first category contains specimens displaying extensive localized corrosion, such as pure Zn, and Zn-0.4Fe, Zn-0.4Cu, and Zn-2.0Ag alloys. The second category, which is the rest of the tested lot, contains those specimens exhibiting uniform corrosion and maintained an intact spherical cross-section. The typical bone–implant interface in each group was selected and examined by SEM and elemental mapping with pure Zn as control (Fig. 6b). Severe, localized corrosion created some pits with depths of tens of microns in pure Zn implant. Degradation products penetrated into the implant and diffused into the surrounding tissue simultaneously. Direct contact between newly formed bone and degradation products was found in some local sites. Compositional analysis demonstrated that Zn and O were the major elements detected in degradation products while Ca and P were rich in new bone tissue. In contrast, the bone-implant interface in Zn-0.4Li alloy was much clear. A shallow, evenly distributed feature with small corroded dimples was observed in this group. As for the Zn-2.0Ag alloy, some locations appeared to be heavily attacked. Corrosion penetrated inside with a depth of over 50 μm. Interestingly, there were some Ag containing second phases in the corrosion interface left uncorroded. Additionally, new bone was found lying on the degradation products as well. Figure 6c presents the chemical compositions of representative regions in Fig. 6b. Region I was the metallic matrix. Degradation products were composed of three different chemical compositions including region II to IV. Region II, as the main components of degradation products, mainly contained C, O, and Zn. Ca and P arose in region III that usually found in the outer layer of degradation products. Region IV had a similar composition to that of the surrounding bone. The Ca/P ratio increased from 0.45 of region III to 1.03 of region IV while Zn content decreased from 13.15 ± 4.21% (*n* = 15) to 5.23 ± 2.77% (*n* = 4). Two inorganic compositions were detected in new bone tissue as well including regions V and VI. The Ca/P ratio of region V and region VI was 1.09 and 1.37, respectively. Meanwhile, Zn content decreased from 8.96 ± 3.00% (*n* = 9) of region V to 1.04 ± 0.52% (*n* = 24) of region VI.

### Osteogenesis and osseointegration

**Fig. 7:**
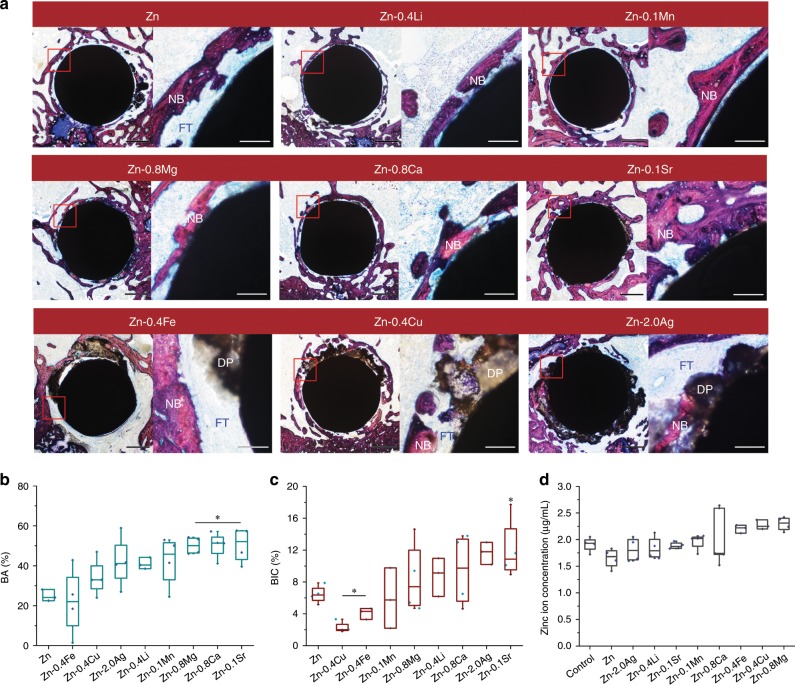
Histological analysis on osteogenesis and osseointegration at 8 weeks. **a** Van Gieson staining of representative cross sections in metaphysis, the magnified region is marked by red rectangle. NB, new bone; DP, degradation products; FT, fibrous tissue. Scale bar, 0.5 mm in low magnification, 500 μm in high magnification. **b** New bone area surrounding the implants, *P < 0.05, compared with Zn (*n* = 4, independent samples). **c** Bone-implant contact ratio, *P < 0.05, compared with Zn (*n* = 4, independent samples). **d** Zinc ion concentration in blood serum at 8 weeks, *P < 0.05, compared with control (*n* = 4, independent samples). *P < 0.05 by one-way ANOVA with Tukey’s post hoc test. For box-whisker plots, box edges correspond to 25th and 75th percentiles, lines inside the box correspond to 50th percentiles, and whiskers include minimum and maximum of all data points. Source data are provided as a Source Data file.

Representative cross-sections of implants at 8 weeks were stained with van Gieson to evaluate their osteogenic and bone integration ability (Fig. 7a). New bone tissue formed around all the implants with different morphologies. Similarly, two categories with distinct features could be observed by histological analysis. In pure Zn, Zn-0.4Fe, Zn-0.4Cu, and Zn-2.0Ag alloys, dark brown degradation products spread into the surrounding tissue with newly formed woven bone dispersed in it. In contrast, larger amounts of new bone tissue could be observed surrounding the implants continuously in Zn-0.4Li, Zn-0.1Mn, Zn-0.8Mg, Zn-0.8Ca, and Zn-0.1Sr alloys. Moreover, the osteocytes in the new bone tissue arranged in a more organized way, indicating a more mature status. The new bone area (BA) adjacent to the implants were analyzed quantitatively (Fig. 7b). Zn-0.1Sr, Zn-0.8Ca, and Zn-0.8Mg alloys exhibited significant higher new bone area than that of pure Zn followed by Zn-0.1Mn, Zn-0.4Li, Zn-2.0Ag, and Zn-0.4Cu alloys. As for osseointegration, direct bone bonding to implants in local sites was found in all the groups. Among them, pure Zn, Zn-0.4Fe, Zn-0.4Cu, and Zn-2.0Ag alloy groups showed thicker intervening fibrous layers than other groups. In contrast, implants were closely integrated with new bone tissue in Zn-0.1Sr, Zn-0.8Ca, Zn-0.4Li, and Zn-0.8Mg alloy groups, showing better bone integration ability. Quantitatively analysis (Fig. 7c) elucidated that Zn-0.1Sr and Zn-2.0Ag alloys exhibited a significant higher bone-implant contact ratio (BIC) than that of pure Zn followed by Zn-0.8Ca, Zn-0.4Li, and Zn-0.8Mg alloys. Unlike Zn-0.1Sr, the new bone tissue in Zn-2.0Ag displayed a shattered feature. The BIC of Zn-0.4Cu and Zn-0.4Fe alloys decreased significantly. The Zn ion concentration in blood serum was collected at 8 weeks and shown in Fig. 7d. No significant difference was found in Zn ion values for all the implant groups compared with the control group.

## Discussion

The mechanical property, biodegradability, and biocompatibility are necessary and sufficient criteria for materials being regarded as biodegradable bone implants. Mg-based orthopedic devices have received extensive studies and been transferred to clinical use successfully. Table 1 compares the major parameters between biodegradable Zn-based materials and Mg-based materials regarding the aspects mentioned above. For mechanical property, the tensile strength of as extruded Mg alloys was usually lower than 350 MPa. Meanwhile, the elongation to failure ranged from 8 to 28%. In contrast, the maximum tensile strength of as extruded Zn alloys in this study reached 520 MPa (Zn-0.4Li alloy). And the elongation to failure was observed from 6% up to 84%. The tensile strength of Zn–Li and Zn–Mg alloy systems were able to exceed 400 MPa easily by hot extrusion or rolling^14^. And the elongation of Zn–Mn, Zn–Cu, and Zn–Ag alloy systems was higher than 30%. Significantly higher values could be seen in compressive strength, microhardness, and elastic modulus as well. An ideal implant should provide adequate strength or at least match the mechanical property of the bone, referred to as biomechanical compatibility. Thus, a material with an excellent combination of high strength and low modulus closer to the bone is desired for bone implants. Biodegradable Zn alloys possess higher modulus than bone that usually varies from 4 to 30 GPa^33^. However, considering the loss of mechanical integrity over time, whether the “stress shielding effect” will be a concern for biodegradable Zn-based bone implants still needs further study. Better mechanical performance enables biodegradable Zn-based materials to have more potential applications in some load-bearing sites compared to biodegradable Mg-based materials.

**Table 1:** Comparison of key properties between Zn-based and Mg-based biodegradable metals.

	Key properties	Zn and Zn alloys	Mg and Mg alloys
Mechanical properties	Yield strength (YS), as extruded (MPa)	126–389	149–293^43,57^
	Ultimate tensile strength (UTS), as extruded (MPa)	167–520	199–350^43,59^
	Compressive yield strength (CYS), as extruded (MPa)	99–457	90–258^60,61^
	Elongation to failure, as extruded (%)	6–84	8–28^34,43^
	Microhardness, as extruded	44–217 (HV)	35–90 (HB)^62^
	Elastic modulus (GPa)	94–110^63^	41–45^33^
Degradability	In vitro degradation rates (SBF, electrochemical test, mm year^−1^)	0.16–1.66	0.45–12.56^20^
	In vitro degradation rates (SBF, static immersion, 30–60 days, mm year^−1^)	0.014–0.03	0.07–1.88^20^
	In vivo degradation rates (Rat femur model, 8–12 weeks, volume reduction, mm year^−1^)	0.13–0.26	0.36–1.58^20^
	Degradation type	General corrosion, localized corrosion, pitting corrosion	Localized corrosion, pitting corrosion^63^
	Major cathodic reaction in neutral physiological environments	Oxygen reduction reaction^35^	Hydrogen evolution reaction^4^
	Major gaseous degradation products	None	Hydrogen^4^
	Major soluble degradation products	Zn^2+^, OH^−^	Mg^2+^, OH^−^
	Major solid degradation products and their solubility^64^	Zn(OH)_2_ (Ksp = 5 × 10^−17^) ZnO (Ksp = 2.5 × 10^−17^) Calcium phosphates	Mg(OH)_2_ (Ksp = 8.9 × 10^−12^) MgO (Ksp = 2.37 × 10^−8^) Calcium phosphates
Biocompatibility	Essential elements on bone metabolism	Yes	Yes
	Human amount (g)^6^	2	25
	Serum concentration (mmol L^−1^)^6^	0.012–0.017	0.73–1.06
	Dietary average daily intake (mg)^53^	8.6	329
	Recommended daily intake (mg)^53^	12–15	280–350
	Beneficial effects on bone^54^	Necessary for bone growth; Prevention of osteopenia and various skeletal abnormalities; Modulate bone turnover by stimulating osteoblast bone formation while inhibiting osteoclast differentiation; Increase bone strength	Necessary for bone growth; Prevention of skeletal fragility, osteoporosis, chronic chondrocalcinosis and myositis ossificans
	Deleterious effects on bone^6^	Hinder bone development at high concentration	None
	IC_50_, osteoblast cells (mmol L^−1^)	0.09^55^	>4.02 (Mg^2+^ concentration in α-MEM)^65^
	IC_50_, endothelial cells (mmol L^−1^)^66^	0.13	66.7
	LD_50_ (mg kg^−1^)^53^	350	5000
	Osteogenesis	Yes	Yes
	Osseointegration	Yes	Yes

Distinct features between Zn and Mg can be observed regarding biodegradability. In general, Mg and its alloys display much higher degradation rates than that of Zn and its alloys both in vitro and in vivo. The degradation rates of Mg and its alloys in rat femur are one to twelve folds faster than that of Zn and its alloys. And the difference is amplified by immersion and electrochemical tests. The bone healing time varies for different fracture sites, and the mechanical support provided by implants should be sustained for 12–24 weeks depending on the clinical conditions^6^. Clinical trials reported the complete degradation of Mg alloy screws at 6 and 12 months^3,4^, which seems to meet the degradation requirement. However, the clinical results of Mg alloy screws are limited in non or low-load bearing sites. Therefore, it is hard to judge whether Mg alloy screws are able to provide sufficient support in high load-bearing sites. In light of this, Zn and its alloys are able to maintain mechanical integrity for longer implantation time and prevent early mechanical failure during service. Degradation of Mg and Zn enables a series of reactions within the physiological environment leading to the formation of gaseous, solid and soluble products. Hydrogen gas is a typical product for Mg and its alloys due to hydrogen evolution reaction during corrosion. Clinical studies reported acceptable outcomes considering gas formation during degradation of Mg alloy bone implants^3,4^. However, excessive hydrogen gas can interfere with the bone healing process, resulting in callus formation and cortical defects^34^. In contrast, X-ray image, Micro-CT, and histology showed no sign of gas formation around Zn and its alloys in this study. Oxygen reduction reaction should be the primary cathodic reaction of zinc in the neutral physiological environment. The dissolved oxygen influences the corrosion of zinc in the pH range from 4 to 12, which applies to most of the potential implantation sites in the human body^35^. Therefore, similar to the biodegradation of iron^36^, the availability of oxygen plays a critical role in the degradation of Zn and its alloys^37^. Hydroxyl ions and metal ions are released during the degradation, leading to the increase of local pH. Corrosion of Mg results in a more pronounced pH increase compared with Zn^9^. The simultaneous increase in pH and metal ion concentrations will lead to the precipitation of oxides and hydroxides. The solubility product constants (*K*_sp_) of Zn(OH)_2_ and ZnO are much lower than that of Mg(OH)_2_ and MgO, indicating a longer dissolution and absorption period in the bone environment. Calcium phosphate is another major product when pH increases. The previous study has reported the formation of calcification matrix, the crystalline calcium phosphate phase with a similar bone-like structure, in the Mg-bone interface. The calcification matrix is further resorbed by osteoclasts and utilized by osteoblasts to form new bone^4,38^. In this study, we identified the product (region IV in Fig. 6b) with a similar chemical composition to that of adjacent new bone. Additionally, a type of new bone tissue (region V in Fig. 6b) with a relatively low Ca/P ratio and high Zn content was detected as well. Thus, it’s reasonable to speculate that the degradation of Zn-based implants should have a similar bone formation aiding mechanism to that of Mg-based implants.

Both Zn and Mg are essential elements on bone metabolism, but their contents in the human body and threshold values for daily intake vary greatly. The human body has a much greater demand for Mg than Zn. Therefore, Zn exhibits a more significant dose-dependent effect on cellular and tissue biocompatibility than Mg at relatively low concentrations. Most of the 100% extracts of Zn and its alloys showed significant cytotoxicity on MC3T3-E1 cells while no cytotoxicity was found in all groups after one-fold dilution. And the Zn ion concentrations in 100% extracts only ranged from 10 to 25 μg mL^−1^. Moreover, endothelial cells are better tolerant of Zn than osteoblast cells. Several studies found a dose-dependent effect of Zn^2+^ on cell viability, proliferation, spreading and migration^19,39,40^. Generally, low concentrations of Zn^2+^ promoted the viability, proliferation, adhesion and migration of osteoblast cells, endothelial cells, and vascular smooth muscle cells, while high concentrations of Zn^2+^ had opposite effects. Mg-based implants have been evaluated by extensive studies in bone environments with different animal models^6,41^. Most of the research reported enhanced new bone formation around the Mg-based implants including promoted local periosteal and endosteal bone formation. As for osseointegration, slow degraded Mg-based implants usually showed direct contact between degradation products and new bone^42–44^. Whereas the presence of fibrous tissue was found in fast degraded ones^45,46^. In contrast, newly formed bone was observed surrounding Zn-based implants at 8 weeks (Fig. 7). And the bone-implant contact ratio (BIC) varied depending on the degradation behavior of implants. Uniform corrosion mode with appropriate degradation rates resulted in improved BIC, while severe localized corrosion provoked a thick fibrotic layer surrounding the implants. Therefore, both Zn-based and Mg-based implants indicate good biocompatibility in bone environments. For Zn-based implants, the concentrations of degradation products should be restricted within a safe threshold to prevent their detrimental effects at high concentrations.

In order to achieve optimized material properties as bone implants, the alloying design strategy for biodegradable Zn alloys is proposed here considering mechanical property, biodegradability, and biocompatibility:

Alloying with Li, Mg, Cu, Ag, and Mn (≤2 wt%) resulted in a significant increase in strength and hardness. Among them, Li displayed the best strengthening effect on Zn followed by Mg. The strengthening mechanism of Li and Mg can be attributed to the grain refinement and intermetallic phases considering the low solubility of these elements in Zn^9,11,47^. The pinning effects caused by a high density of fine dispersive LiZn_4_ precipitates (10–30 nm) played a major role in increasing the strength of Zn–Li alloys^48^. Naturally, strengthening always comes with a reduction in ductility. However, no detrimental effect on ductility was found after adding Cu and Ag. And alloying with Mn resulted in a superb elongation compared with other Zn alloys. Apart from the solid solution and refined grain, tensile twin was a key factor affecting the mechanical property of as-extruded Zn–Mn alloys^49^. Although Ca and Sr showed no strengthening effect on Zn in this study, an increased tensile strength of Zn–Ca, Zn–Sr, and Zn–Fe alloys with higher contents were observed elsewhere^16,50^.

Alloying with selected elements here increased the degradation rates of Zn to varying degrees. Galvanic corrosion was observed in all binary Zn alloys with intermetallic phases. Zn acted as the anode in Zn–Fe, Zn–Cu, and Zn–Ag alloys while Zn was the cathode in other alloys. Among them, Cu and Ag showed the most significant effect on accelerating degradation. Adding Fe, Cu, and Ag resulted in severe localized corrosion in the rat femur. Whereas uniform corrosion was dominant in other binary alloy systems. According to the bone healing time for different fractures, bone implants are suggested to maintain mechanical integrity for at least 3–6 months. The volume loss of pure Zn and its alloys was less than 10% at 8 weeks, indicating the complete degradation of them may need at least 20 months based on a linear degradation trend. Therefore, a faster degradation rate should be more appropriate. However, efforts should be made to establish a more accurate and reasonable standard for degradation through animal or even clinical tests. Apart from this, it is critical to control the concentration of degradation products within a beneficial range during the degradation of Zn-based implants. To achieve the accelerated and controlled degradation simultaneously, elements with lower electrode potentials should be considered preferentially as alloying elements to Zn. The second phases formed between Zn and these elements will act as anodes and, consequently, creating galvanic couples. As a result, accelerating the overall corrosion while inhibiting the corrosion of the Zn matrix can be expected.

Alloying with Li and Mg exhibited the most distinct effect in improving the cytocompatibility of Zn. Moreover, adding Ca and Sr with appropriate content was able to eliminate the toxicity of Zn as well. More importantly, enhanced new bone formation and bone integration ability were observed after alloying with Mg, Ca, Sr, and Li. Among them, Sr showed the most significant effect in improving the performance of Zn. It is well known that Ca and Mg are essential elements that play pivotal roles in bone health^21,22^. Sr stimulated osteoblast replication and differentiation and increased cell survival under stress^51,52^. Li improved bone mass in mice and enhanced bone formation via activation of the canonical Wnt pathway^25^. And maintenance therapy with lithium carbonate enhanced bone mass^26^. Therefore, elements that play essential or beneficial roles in bone metabolism can be regarded as potential alloying elements. Metallic elements (K, Na, Ba, and Mo) and nonmetallic elements (O, P, S, Si, and Se) can be added into Zn to develop novel Zn alloy systems for better biocompatibility in the future^53–55^.

**Fig. 8:**
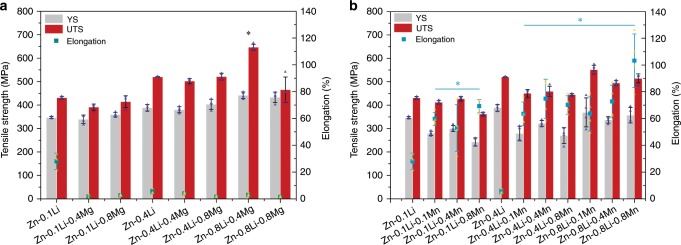
Mechanical properties of biodegradable ternary Zn alloys. **a** Zn–Li–Mg alloy, *P < 0.05, compared with Zn-0.4Li alloy. **b** Zn–Li–Mn alloy, *P < 0.05, compared with Zn-0.1Li alloy. Error bars indicate mean ± standard deviation (*n* = 4, independent samples). *P < 0.05 by one-way ANOVA with Tukey’s post hoc test. Source data are provided as a Source Data file.

Based on the above discussion, Zn–Li, Zn–Mg, Zn–Ca, and Zn–Sr alloy systems displayed the most desirable comprehensive properties for developing Zn-based bone implants. Material optimization including multi-alloy systems and composites and device design can be further developed based on these alloy systems. Among them, the Zn–Li alloy system exhibited the greatest potential to be used for load-bearing applications. Therefore, we further designed ternary Zn–Li–X alloys for optimization. Mg and Mn were added into Zn–Li alloy for the purpose of strengthening the matrix or improving the ductility, respectively. As shown in Fig. 8, as-extruded Zn-0.8Li-0.4Mg alloy reached a UTS of 646.69 ± 12.79 MPa (*n* = 4), which is the highest tensile strength reported for Zn alloys according to latest review paper^14^. As for Zn–Li–Mn alloys, surprisingly high elongation was found after adding Mn compared with Zn–Li alloys. Zn-0.8Li-0.8Mn alloy possessed an elongation to failure up to 103.27 ± 20% (*n* = 4) while maintaining a UTS of 514.43 ± 19.36 MPa (*n* = 4). The outstanding mechanical properties of Zn–Li–Mg and Zn–Li–Mn alloys are comparable to clinically used pure Ti and 316 stainless steel.

**Fig. 9:**
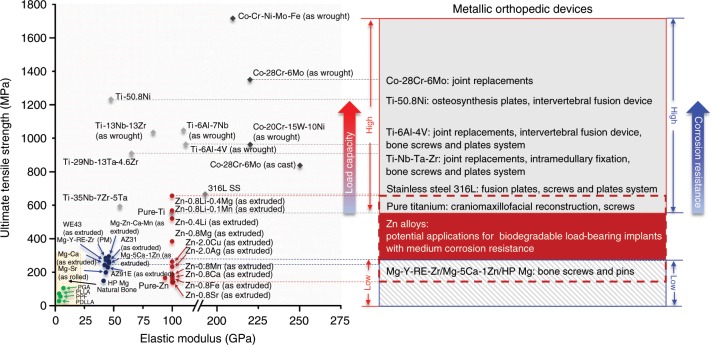
Mechanical properties of biodegradable and non-biodegradable materials for orthopedic devices and their clinical applications. (Ti-based alloys, Co-Cr alloys and 316 L stainless steel^7,8,56^, biodegradable Mg-based alloys^3,4,41,43,45,57^, biodegradable polymers^1,58^).

Figure 9 illustrates the mechanical properties and clinical applications of currently used materials for orthopedic devices. Cobalt chromium alloys, 316 L stainless steel, and titanium-based alloys are major non-degradable materials for load-bearing applications due to their high strength, superior corrosion resistance in body environment and excellent biocompatibility. Among them, titanium alloys are fast emerging as the first choice for the majority of applications due to its high immunity to corrosion, low modulus, and high capacity to join with bone. Commercial pure Ti and Ti-6Al-4V are the most commonly used titanium materials, which cover joint replacements, intervertebral fusion devices, craniomaxillofacial reconstruction, and bone screw and plate systems, from high load-bearing to low load-bearing applications. To avoid stress shielding and secondary surgery, biodegradable materials including polymers and metals have been developed for orthopedic devices. Polyglycolide (PGA), polylactide (PLA), and poly (l-lactic acid) (PLLA) are FDA approved polymer products with similar mechanical properties to cancellous bone. However, they are only intended for applications like soft tissue graft fixation and meniscus repair due to their low strength. In contrast to polymers, Mg-based materials possess higher strength and modulus that are close to cortical bone. Mg-based materials advanced rapidly in recent years with two material products (MgYReZr and MgZnCa alloys) approved by Conformité Européene (CE) and the Korea Food and Drug Administration (KFDA), respectively. They are fabricated into bone screws and pins indicated for intra-articular and extra-articular fractures of small bones and bone fragments. Therefore, both polymers and Mg-based materials are incapable of high load-bearing applications as a result of their insufficient mechanical strength. In contrast, the mechanical strength of Zn alloys falls in a wide range, from the value of pure Mg to the value of commercial pure Ti and 316 stainless steel. The promising mechanical performance of Zn alloys can motivate scientists and clinicians to consider using biodegradable Zn-based implants in not only low load-bearing sites but also some of the high load-bearing sites and extend the clinical applications of biodegradable implants.

In summary, our work evaluated binary Zn alloys as biodegradable bone implants comprehensively by both in vitro and in vivo experiments with regard to mechanical property, biodegradability, and biocompatibility. Our findings have significant implications for understanding the degradation behavior and biological responses of Zn-based alloys in bone environments. This study lays the groundwork for future research into the design strategy of biodegradable Zn-based alloys specifically as bone implants and directs their clinical prospects. Zn-based alloys have immense potential to play a pivotal role in biodegradable bone implants for load-bearing applications.

## Methods

### Material preparation

Pure Zn and Zn alloys were fabricated by Hunan rare earth metal material research institute. The analyzed chemical compositions of nominal binary and ternary Zn alloys were given in Supplementary Tables 1 and 2. For Mg, Ca, Sr, Li, Mn and Fe, 0.1, 0.4, and 0.8 wt% of alloying elements were added due to their limited solubility in Zn. 0.4, 0.8, and 2.0 wt% of Cu and Ag were added based on their relatively high solubility in Zn. The material ingots were homogenized at 350 °C for 48 h followed by water quenching. Then, the ingots were hot extruded at 260 °C with an extrusion ratio of 36:1. The as-extruded pure Zn and Zn alloys were cut into square samples (10 × 10 × 1 mm^3^) perpendicular to the extrusion direction for microstructure, corrosion and cytocompatibility tests. All samples were grounded to 2000 grit with SiC, followed by ultrasonically cleaning in acetone, absolute ethanol and distilled water. Samples were sterilized by ultraviolet-radiation for at 4 h for each side before cell tests.

### Microstructure characterization

Samples were further grounded to 7000 grit and polished by 0.1 μm diamond paste, then cleaned in distill water. All samples were etched with a 4% HNO_3_/alcohol solution. A SEM (Hitachi S-4800, Japan) equipped with energy dispersive spectrometry (EDS) was utilized for microstructure observation and composition analysis. X-ray diffractometer (XRD, Rigaku DMAX 2400, Japan) using CuKα was operated at 40 kV and 100 mA to identify the phase composition of samples with scanning range from 10° to 90° at a scan rate of 2° min^−1^ and step of 0.02°.

### Mechanical tests

Samples for tensile tests and compressive tests were machined along the extrusion direction according to ASTM-E8-04a and ASTM-E9-89 and carried out in a universal material test machine (Instron 5969, USA) at room temperature. For tensile and compressive tests, the displacement rates were 1 × 10^−4^ s^−1^ and 2 × 10^−4^ s^−1^, respectively. The yield strength was determined as the stress at which the 0.2% plastic deformation occurred. The maximum stress before 50% compressive strain was defined as ultimate compressive strength. Microhardness test was adopted by a microhardness tester (SHIMADZUHMV-2t) measuring Vickers hardness with 0.1 kN loading force and 15 s dwell time. An average of at least five measurements was taken for each group.

### Electrochemical tests

The electrochemical tests were conducted with an electrochemical working station (Autolab, Metrohm, Switzerland) at 37 °C in SBF solution (NaCl 8.035 g L^−1^, NaHCO_3_ 0.355 g L^−1^, KCl 0.25 g L^−1^, K_2_HPO_4_·3H_2_O 0.231 g L^−1^, MgCl_2_·6H_2_O 0.311 g L^−1^, HCl (36–38%) 39 mL L^−1^, CaCl_2_ 0.292 g L^−1^, Na_2_SO_4_ 0.072 g L^−1^, Tris 6.118 g L^−1^, pH 7.4). A three-electrode cell with counter electrode made of platinum and a saturated calomel electrode (SCE) as the reference electrode was used. The open-circuit potential (OCP) was monitored for 5400 s for each sample. Potentiodynamic polarization was carried out at a scan rate of 1 mV s^−1^ ranging from −500 to 500 mV (vs. OCP), and with a test area of 0.2826 cm^2^. Corrosion potential (*E*_corr_) and corrosion current density (*i*_corr_) were calculated by linear fit and Tafel extrapolation. An average of at least five measurements was taken for each group.

### Immersion tests

Samples were immersed in SBF solutions at 37 °C for 30 days with a solution to area ratio of 20 mL cm^−2^ according to ASTM-G31-72. The pH value was monitored by a pH meter (Mettler Five Easy pH FE20K) and the solution was refreshed every 48 h. After immersion, samples were rinsed by distill water and dried in air. The corrosion morphology before and after removal of corrosion products was observed by SEM. A solution containing 200 g L^−1^ CrO_3_ was used for cleaning the corrosion products. The corrosion rates of samples were calculated based on the weight loss according to the equation: *C* = Δ*m* *ρ*^−1^ *A*^−1^ t^−1^, where *C* is the corrosion rate in mm year^−1^, *Δm* is the weight loss, *ρ* is the density of the material, *A* is the initial implant surface area, and *t* is the implantation time. An average of at least five measurements was taken for each group.

### Cytocompatibility

Osteoblast precursor cell line (MC3T3-E1, ATCC CRL-2594™) and human umbilical vein endothelial cells (HUVECs, ATCC CRL-1730™) were adopted to evaluate the cytotoxicity of pure Zn and binary Zn alloys. MC3T3-E1 and HUVEC cells were cultured in alpha-minimum essential medium (MEM) and Dulbecco’s modified Eagle’s medium (DMEM) with 10% fetal bovine serum (FBS), 100 U mL^−1^ penicillin and 100 μg mL^−1^ streptomycin at 37 °C in a humidified atmosphere of 5% CO_2_. Extracts were prepared by using culture medium with a surface area to medium ratio of 1.25 mL cm^−2^ at 37 °C in a humidified atmosphere of 5% CO_2_ for 24 h. The supernatant fluid was withdrawn, centrifuged and kept at 4 °C prior to use. Cells were incubated in 96-well plates at a density of 3 × 10^4^ cells mL^−1^ in each well for 24 h to allow attachment. The culture medium was then replaced by 100 μL 100 and 50% sample extracts. The culture medium was used as negative control and culture medium with 10% dimethyl sulfoxide (DMSO, Invitrogen, USA) as positive control. After 1, 2, and 4 days’ incubation, the extracts were replaced by fresh culture medium, 10 μL Cell Counting Kit-8 (CCK-8, Dojindo, Kumamoto, Japan) was added into each well and incubated at 37 °C in a humidified atmosphere of 5% CO_2_ for 1 h. The spectrophotometrical absorbance of mediums were measured at 450 nm using a microplate reader (Bio-RAD680). An average of at least five measurements was taken for each group. For cytoskeleton and cell spreading, sterilized samples were put in 24-well plates. Five hundred microliter cell suspension were added into the samples at a density of 8 × 10^4^ cells mL^−1^ and incubated for 12 h. cells were then washed with PBS, fixed using 4% (w v^−1^) paraformaldehyde for 10 min and permeabilized with 0.1% (v v^−1^) Triton X-100 (Sigma) for 7 min. Afterwards, 1 mg mL^−1^ DAPI (Sigma) and 1.0% (v v^−1^) FITC-phalloidin (Sigma) were used to stain the nuclei and cellular actin for 5 min and 30 min, respectively. Cells were washed by PBS to remove the residue dye and viewed under an inverted confocal laser scanning microscope (CLSM, Leica TCS SP5, Germany).

### Surgical procedure

Implants of pure Zn and binary Zn alloys (Φ1.6 × 15 mm) were machined from the as-extruded samples. A rat femur model was used and the surgical procedures were conducted under sterile conditions. Intraperitoneal injection of ketamine (10 mg kg^−1^) and 2% xylazine (5 mg kg^−1^) were used to anesthetize the rats. Each rat was immobilized with the knee joint in maximally flexed position and the right hind limb was shaved and depilated. A 15 mm long incision was made longitudinally along the lateral side of the humerus to dislocate the knee joint. With the knee in flexion, a cylindrical hole (1.6 mm in diameter) was drilled in the center of the femoral condyle parallel to the long axis of the femur. After the bone cavity was washed with normal saline, a metal implant was inserted into the right femur, and, subsequently, the wound was closed carefully. Each rat received only one implant. At least six implants of each alloy type were tested. At least fifty-four male Sprague Dawley rats aged 3 months and weighed by an average of 200 g were randomized to either group. After surgery, the rats were housed in ventilated rooms and given access to water and food. At 8 weeks, the rats were sacrificed and the right femurs were explanted and fixed in 10% neutral formalin buffer for Micro-CT and histological analysis. X-ray scan was performed post surgically and at 8 weeks. The Zn ion concentrations in blood serum were measured by inductively coupled Plasma optical emission spectroscope (ICP-OES, iCAP6300, Thermo). All the animal procedures have complied with relevant ethical regulations for animal research and were approved by the Animal Ethical Committee at the Ninth People’s Hospital affiliated to Shanghai Jiaotong University, School of Medicine (Shanghai, China).

### Micro-CT analysis

A Skyscan 1172 Micro-CT system (Bruker Micro-CT N.V., Kontich, Belgium) was adopted to analyze the implants. Explanted rat femurs were examined with a 20 μm resolution protocol (100 kV, Al + Cu filter, 0.6° rotation step, frame averaging of 2°, 360° rotation). The CT images were reconstructed by using Skyscan NRecon software and further analyzed by CTAn, CTVol, and CTVox software to produce 3D images and volume loss data. The degradation rate, DR, was calculated based on the equation:

1 $${\mathrm{DR}} = \frac{{V_0 - V_{\mathrm{t}}}}{{{\mathrm{At}}}},$$

where *V*_0_ is the volume of the implant before implantation, *V*_t_ is the volume of the implant at the designated implantation time interval. *t* is the implantation time and *A* is the initial implant surface area.

### Cross sectional analysis

Hard tissue blocks were cut to produce sections of 1 mm thickness. Cross section samples were prepared by grinding with 7000 grit SiC paper and polished with 0.1 μm diamond paste. Cross sections in the femoral condyle were used for examination. At least five sections were produced in each group. The polished samples were coated with a thin layer of gold before analyzing by SEM equipped with EDS.

### Histological preparation and histomorphometric analysis

After fixation, the implants were rinsed in water, dehydrated in ethanol, cleared in xylene and embedded in methyl methacrylate. The histological sections were generated perpendicular to the long axis of the implants around the femoral condyle. At least six sections were produced in each group for analysis. Sections were grinded to 100 μm thickness, polished and stained with van Gieson’s Picro-fuschin. The specimens were observed under a high-quality microscope (Olympus CKX41, Japan). To quantify bone-to-implant contact (BIC) and bone area (BA), the osseointegrated implant surface and bone area within a ring of 100 μm around the implant were assessed. The BIC represents the available implant perimeter in contact with bone normalized over the implant perimeter length. BA was defined as a ratio of bone area to total area extending 100 μm from the implant.

### Statistical analysis

The data were evaluated by one-way analysis of variance (ANOVA) followed by post hoc Tukey’s multiple comparison test. The data were presented as mean ± standard deviation (*n* ≥ 3, independent samples) and a difference of *P < 0.05 was considered significant.

### Reporting summary

Further information on research design is available in the Nature Research Reporting Summary linked to this article.

## Supplementary information

Supplementary Information

Reporting Summary

## Data Availability

The source data underlying Figs. 1b, c, 2a–c, 3b, c, 4a, 5b, c, 6c, 7b–d, 8a, b and Supplementary Figs. 1, 3a–c and Supplementary Table 3 are provided as a Source Data file. Additional data related to this paper may be requested from the authors.

## Source data

Source Data
