# Supplementary material for: Alloying design of biodegradable zinc as promising bone implants for load-bearing applications
Source: Nat Commun. 2020 Jan 21;11:401. doi: 10.1038/s41467-019-14153-7 (PMC6972918; doi:10.1038/s41467-019-14153-7)
Supplement: Supplementary file 1 — Supplementary Information [file 41467_2019_14153_MOESM1_ESM.pdf]

Supplementary Information

Alloying design of biodegradable zinc as promising bone implants for load-bearing applications

Yang et al.

Supplementary Figures

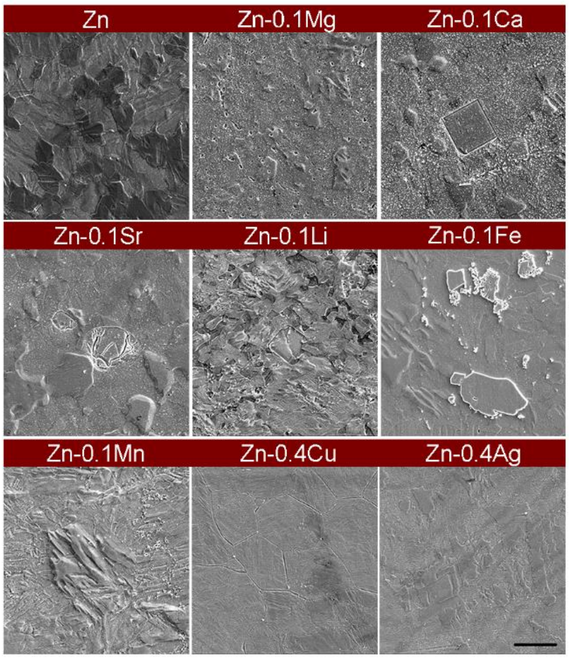

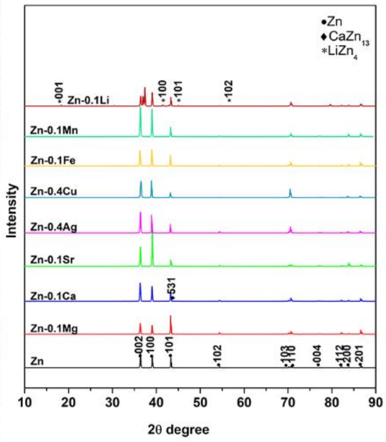

Supplementary Figure 1. SEM images and X-ray diffraction (XRD) of pure Zn and binary Zn alloys at the lowest alloy contents. Scale bar, 10 µm. Source data are provided as a Source Data file.

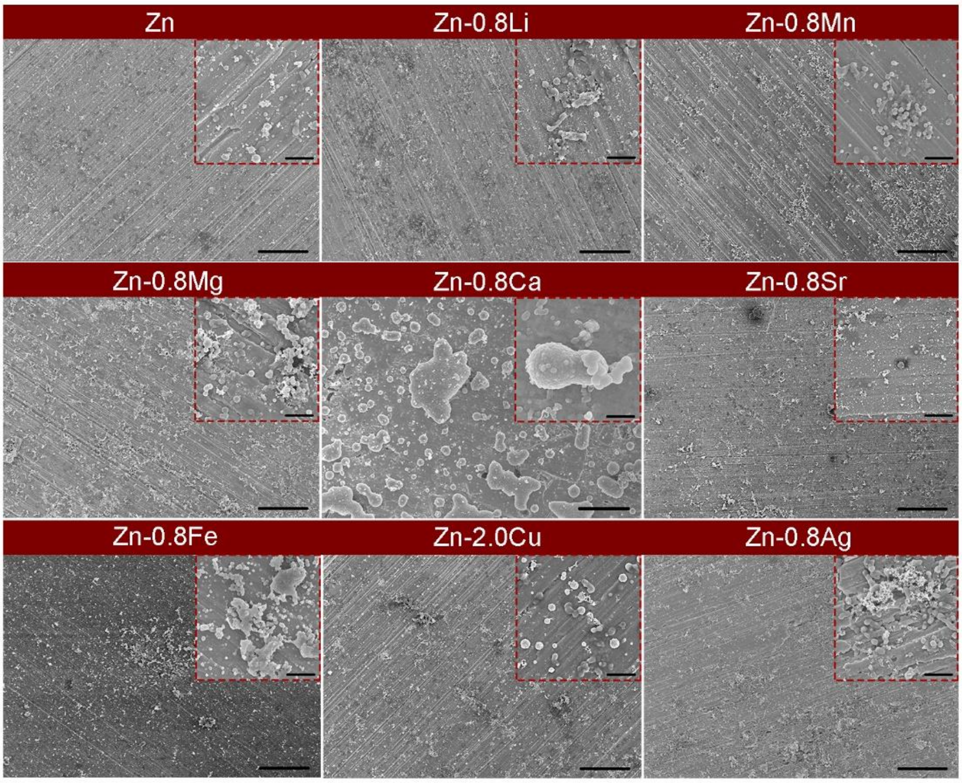

Supplementary Figure 2. Corrosion morphology of selected materials after immersion in SBF for 30 days. Scale bar, 20 μm in low magnification, 2 μm in the inserts.

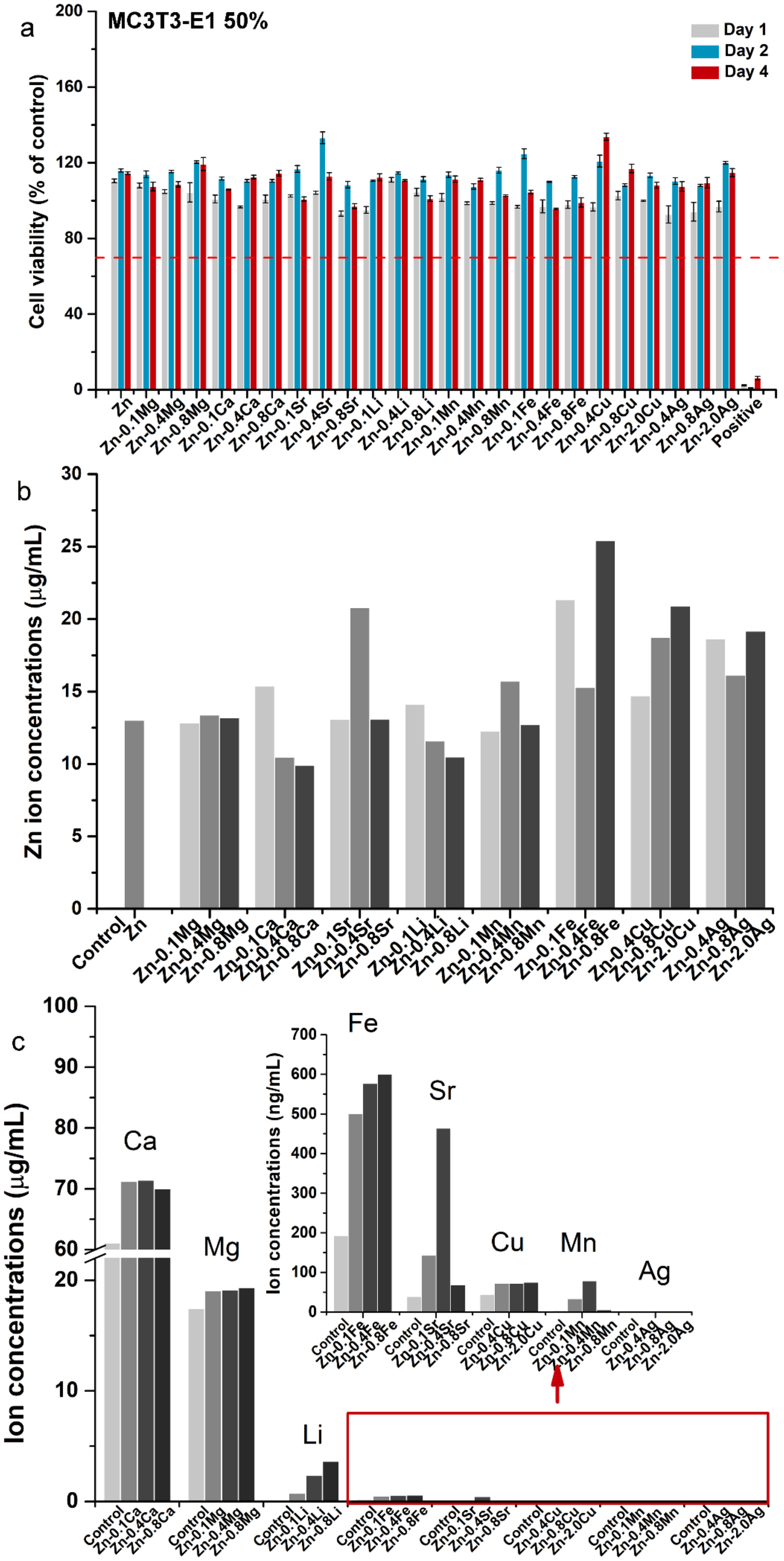

a
MC3T3-E1 50%
Day 1
Day 2
Day 4
Cell viability (% of control)
Zn
Zn-0.1Mg
Zn-0.4Mg
Zn-0.8Mg
Zn-0.1Ca
Zn-0.4Ca
Zn-0.8Ca
Zn-0.1Sr
Zn-0.4Sr
Zn-0.8Sr
Zn-0.1Li
Zn-0.4Li
Zn-0.8Li
Zn-0.1Mn
Zn-0.4Mn
Zn-0.8Mn
Zn-0.1Fe
Zn-0.4Fe
Zn-0.8Fe
Zn-0.4Cu
Zn-0.8Cu
Zn-2.0Cu
Zn-0.4Ag
Zn-0.8Ag
Zn-2.0Ag
Positive
b
Zn ion concentrations (μg/mL)
Control
c
Ion concentrations (μg/mL)
Ca
Mg
Li
Fe
Sr
Cu
Mn
Ag
Ion concentrations (ng/mL)

Supplementary Figure 3. Cytocompatibility of pure Zn and binary Zn alloys. **a** Cell viability of MC3T3-E1 cells cultured with 50% extracts. **b** Zn ion concentrations and **c** alloying element ion concentrations in 100% extracts. Error bars indicate mean ± standard deviation (n=3, independent samples). *$P<0.05$ by one-way ANOVA with Tukey's post hoc test, compared with Zn. The red dashed line indicates the cut-off between non-toxic and toxic responses according to ISO 10993-5. Source data are provided as a Source Data file.

Supplementary Tables

Supplementary Table 1 Chemical composition of pure Zn and binary Zn alloys

Materials	Composition (wt. %)	
	Alloy contents	Zn
Pure Zn	-	99.99
Zn-0.1Sr	0.11	Bal.
Zn-0.4Sr	0.42	Bal.
Zn-0.8Sr	0.77	Bal.
Zn-0.1Ca	0.14	Bal.
Zn-0.4Ca	0.38	Bal.
Zn-0.8Ca	0.76	Bal.
Zn-0.1Mg	0.14	Bal.
Zn-0.4Mg	0.43	Bal.
Zn-0.8Mg	0.77	Bal.
Zn-0.1Li	0.13	Bal.
Zn-0.4Li	0.44	Bal.
Zn-0.8Li	0.79	Bal.
Zn-0.1Fe	0.11	Bal.
Zn-0.4Fe	0.43	Bal.
Zn-0.8Fe	0.81	Bal.
Zn-0.1Mn	0.11	Bal.
Zn-0.4Mn	0.37	Bal.
Zn-0.8Mn	0.79	Bal.
Zn-0.4Ag	0.43	Bal.
Zn-0.8Ag	0.82	Bal.
Zn-2.0Ag	1.92	Bal.
Zn-0.4Cu	0.45	Bal.
Zn-0.8Cu	0.85	Bal.
Zn-2.0Cu	1.98	Bal.

Supplementary Table 2 Chemical composition of ternary Zn alloys

Materials	Composition (wt. %)			
	Li	Mg	Mn	Zn
Zn-0.1Li-0.4Mg	0.11	0.38	-	Bal.
Zn-0.1Li-0.8Mg	0.10	0.82	-	Bal.
Zn-0.4Li-0.4Mg	0.38	0.39	-	Bal.
Zn-0.4Li-0.8Mg	0.39	0.78	-	Bal.
Zn-0.8Li-0.4Mg	0.78	0.39	-	Bal.
Zn-0.8Li-0.8Mg	0.79	0.80	-	Bal.
Zn-0.1Li-0.1Mn	0.11	-	0.12	Bal.
Zn-0.1Li-0.4Mn	0.10	-	0.42	Bal.
Zn-0.1Li-0.8Mn	0.10	-	0.78	Bal.
Zn-0.4Li-0.1Mn	0.38	-	0.12	Bal.
Zn-0.4Li-0.4Mn	0.38	-	0.38	Bal.
Zn-0.4Li-0.8Mn	0.37	-	0.81	Bal.
Zn-0.8Li-0.1Mn	0.76	-	0.12	Bal.
Zn-0.8Li-0.4Mn	0.80	-	0.38	Bal.
Zn-0.8Li-0.8Mn	0.81	-	0.77	Bal.

Supplementary Table 3 Electrochemical parameters for pure Zn and binary Zn alloys in SBF solution

Materials	I_{corr} (μA·cm^{-2})	E_{corr} (V)	Corrosion rate (mm·year^{-1})
Pure zinc	8.523 (1.077)	-0.995 (0.004)	0.254 (0.032)
Zn-0.1Mg	16.040 (3.683)	-1.087 (0.060)	0.408 (0.129)
Zn-0.4Mg	13.470 (1.296)	-1.118 (0.002)	0.401 (0.039)
Zn-0.8Mg	15.245 (1.816)*	-1.022 (0.072)	0.454 (0.054)*
Zn-0.1Ca	17.837 (5.663)*	-1.185 (0.024)	0.531 (0.169)*
Zn-0.4Ca	7.461 (1.725)	-0.983 (0.005)	0.222 (0.051)
Zn-0.8Ca	10.489 (0.114)	-1.202 (0.014)	0.312 (0.004)
Zn-0.1Sr	7.050 (0.895)	-1.017 (0.013)	0.210 (0.027)
Zn-0.4Sr	20.562 (2.592)*	-1.134 (0.007)	0.612 (0.077)*
Zn-0.8Sr	27.817 (3.234)*	-1.141 (0.013)	0.828 (0.096)*
Zn-0.1Li	15.365 (1.614)*	-1.038 (0.009)	0.457 (0.048)*
Zn-0.4Li	11.339 (3.290)	-1.025 (0.023)	0.338 (0.098)
Zn-0.8Li	30.407 (2.750)*	-1.112 (0.007)	0.905 (0.082)*
Zn-0.1Mn	5.374 (0.259)*	-0.951 (0.049)	0.160 (0.008)*
Zn-0.4Mn	7.220 (3.034)	-0.961 (0.028)	0.190 (0.122)
Zn-0.8Mn	7.243 (0.753)	-0.984 (0.008)	0.108 (0.011)
Zn-0.1Fe	49.376 (17.381)*	-1.141 (0.007)	1.470 (0.518)*
Zn-0.4Fe	9.304 (1.311)	-1.043 (0.003)	0.277 (0.039)
Zn-0.8Fe	55.591 (13.755)*	-1.126 (0.011)	1.656 (0.409)*
Zn-0.4Cu	7.652 (0.872)	-0.976 (0.022)	0.228 (0.026)
Zn-0.8Cu	37.153 (7.213)*	-1.103 (0.003)	1.106 (0.214)*
Zn-2.0Cu	31.518 (4.012)*	-1.108 (0.004)	0.938 (0.119)*
Zn-0.4Ag	35.625 (4.489)*	-1.047 (0.083)	1.061 (0.133)*
Zn-0.8Ag	45.991 (4.195)*	-1.095 (0.005)	1.369 (0.124)*
Zn-2.0Ag	17.291 (2.630)*	-1.061 (0.091)	0.515 (0.078)*

Numbers in the brackets represent the standard deviation (n=3, independent samples), *$P<0.05$ by one-way ANOVA with Tukey's post hoc test, compared with pure Zn. Source data are provided as a Source Data file.
