# Supplementary material for: Alloying design of biodegradable zinc as promising bone implants for load-bearing applications
Source: Nat Commun. 2020 Jan 21;11:401. doi: 10.1038/s41467-019-14153-7 (PMC6972918; doi:10.1038/s41467-019-14153-7)
Supplement: Supplementary file 2 — Reporting Summary [file 41467_2019_14153_MOESM2_ESM.pdf]

nature research

Corresponding author(s): Yufeng Zheng

Last updated by author(s): Nov 19, 2019

Reporting Summary

Nature Research wishes to improve the reproducibility of the work that we publish. This form provides structure for consistency and transparency in reporting. For further information on Nature Research policies, see Authors & Referees and the Editorial Policy Checklist .

Statistics

For all statistical analyses, confirm that the following items are present in the figure legend, table legend, main text, or Methods section.

n/a	Confirmed	
☐	☒	The exact sample size (*n*) for each experimental group/condition, given as a discrete number and unit of measurement
☐	☒	A statement on whether measurements were taken from distinct samples or whether the same sample was measured repeatedly
☐	☒	The statistical test(s) used AND whether they are one- or two-sided<br>*Only common tests should be described solely by name; describe more complex techniques in the Methods section.*
☒	☐	A description of all covariates tested
☒	☐	A description of any assumptions or corrections, such as tests of normality and adjustment for multiple comparisons
☐	☒	A full description of the statistical parameters including central tendency (e.g. means) or other basic estimates (e.g. regression coefficient) AND variation (e.g. standard deviation) or associated estimates of uncertainty (e.g. confidence intervals)
☐	☒	For null hypothesis testing, the test statistic (e.g. *F*, *t*, *r*) with confidence intervals, effect sizes, degrees of freedom and *P* value noted<br>*Give P values as exact values whenever suitable.*
☒	☐	For Bayesian analysis, information on the choice of priors and Markov chain Monte Carlo settings
☒	☐	For hierarchical and complex designs, identification of the appropriate level for tests and full reporting of outcomes
☒	☐	Estimates of effect sizes (e.g. Cohen's *d*, Pearson's *r*), indicating how they were calculated

Our web collection on statistics for biologists contains articles on many of the points above.

Software and code

Policy information about availability of computer code

Data collection	OriginPro 9, Microsoft Excel, Microsoft PowerPoint, Jade 6, Nova 1.11, ImageJ, Adobe Photoshop CS6, and Adobe Illustrator CS6 was used for performing data collection and generating figures.
Data analysis	OriginPro 9, Microsoft Excel, Microsoft PowerPoint, Jade 6, Nova 1.11, ImageJ, Adobe Photoshop CS6, and Adobe Illustrator CS6 was used for performing data analysis and generating figures.

For manuscripts utilizing custom algorithms or software that are central to the research but not yet described in published literature, software must be made available to editors/reviewers. We strongly encourage code deposition in a community repository (e.g. GitHub). See the Nature Research guidelines for submitting code & software for further information.

Data

Policy information about availability of data

All manuscripts must include a data availability statement. This statement should provide the following information, where applicable:

- Accession codes, unique identifiers, or web links for publicly available datasets
- A list of figures that have associated raw data
- A description of any restrictions on data availability

The source data underlying Figs. 1b, 1c, 2a-2c, 3b, 3c, 4a, 5b, 5c, 6c, 7b-7d, 8a, 8b and Supplementary Figures 1, 3a-3c and Supplementary Table 3 are provided as a Source Data file. Additional data related to this paper may be requested from the authors.

Field-specific reporting

Please select the one below that is the best fit for your research. If you are not sure, read the appropriate sections before making your selection.

Life sciences study design

All studies must disclose on these points even when the disclosure is negative.

Sample size	Each finding was confirmed with minimum necessary number such as 1-6 replicates for each experiments.
Data exclusions	In order to reduce the deviation, the maximum and minimum values are removed when analyzing mechanical property data, degradation data, cell viability data and animal test data.
Replication	All experiments were performed with independent replicates as described in the figure legends.
Randomization	Randomization was performed blindly among animals.
Blinding	N/A

Reporting for specific materials, systems and methods

We require information from authors about some types of materials, experimental systems and methods used in many studies. Here, indicate whether each material, system or method listed is relevant to your study. If you are not sure if a list item applies to your research, read the appropriate section before selecting a response.

Materials & experimental systems

n/a	Involved in the study
☒	☐ Antibodies
☐	☒ Eukaryotic cell lines
☒	☐ Palaeontology
☐	☒ Animals and other organisms
☒	☐ Human research participants
☒	☐ Clinical data

Methods

n/a	Involved in the study
☒	☐ ChIP-seq
☒	☐ Flow cytometry
☒	☐ MRI-based neuroimaging

Eukaryotic cell lines

Policy information about cell lines

Cell line source(s)	Osteoblast precursor cell line, human umbilical vein endothelial cells
Authentication	MC3T3-E1 ATCC CRL-2594TM, HUVECs ATCC CRL-1730TM
Mycoplasma contamination	N/A
Commonly misidentified lines (See ICLAC register)	N/A

Animals and other organisms

Policy information about studies involving animals; ARRIVE guidelines recommended for reporting animal research

Laboratory animals	At least fifty-four male Sprague Dawley rats aged 3 months and weighed by an average of 200 g were randomized to either group.
Wild animals	N/A
Field-collected samples	N/A
Ethics oversight	All the animal procedures have complied with relevant ethical regulations for animal research and were approved by the Animal Ethical Committee at the Ninth People's Hospital affiliated to Shanghai Jiaotong University, School of Medicine (Shanghai, China).

Note that full information on the approval of the study protocol must also be provided in the manuscript.
